# Manufacturing and characterization of innovative lightweight wooden furniture from polystyrene core wood sandwich panels

**DOI:** 10.1038/s41598-025-11044-4

**Published:** 2025-09-30

**Authors:** Orhan Kelleci, Süheyla Esin Köksal, Eser Sözen, Kadir Kayahan, Deniz Aydemir

**Affiliations:** 1 Mudurnu Sureyya Astarcı Vocational School, Forestry and Forest Product department, Bolu Abant Izzet Baysal University, 14100 Bolu, Türkiye; 2 Forestry Faculty, Forestry Industry Engineering department, Bartin University, 74100 Bartın, Türkiye; 3 Bartin Vocational School, Material and Material Processing Technologies department, Bartin University, 74100 Bartın, Türkiye

**Keywords:** Lightweight, Smart furniture, Wood-based sandwich structures, Polystyrene core, Engineered wood panels, Composites, Environmental economics, Forestry

## Abstract

The increasing demand for lightweight and sustainable furniture has highlighted the need for innovative materials with improved performance characteristics. In this study, a light wood sandwich panel (HM) was produced by incorporating polystyrene cores, and lightweight furniture was manufactured using these panels. The primary objective was to develop alternative lightweight materials for furniture production and to perform a comparative analysis of the physical and mechanical properties of HM, medium density fiberboard (MDF), and particleboard (PB). Physical and mechanical characterization tests, including swelling, water absorption, internal bonding strength, screw withdrawal force, and modulus of rupture, were conducted on HM boards and compared with MDF and PB. Results showed that HM boards were 77% lighter than MDF and 16% lighter than PB. HM exhibited the lowest swelling and water absorption values and the highest internal bonding strength among the tested materials. However, the screw withdrawal force and modulus of rupture of HM were lower than those of MDF and PB. Despite these limitations, HM panels demonstrated superior lightweight characteristics and high water resistance. Further improvements in mechanical properties are possible through the use of stronger adhesives and fastening techniques. Overall, the findings indicate that HM panels offer a promising alternative for lightweight furniture production, potentially reducing wood consumption and production costs, while creating more ergonomic products.

## Introduction

Natural disasters, exemplified by earthquakes, are unforeseen and abrupt events that have the potential to cause substantial damage to living spaces^1^. Indoor furniture poses a risk of toppling and shifting during an earthquake, threatening both life and property. Toppled furniture can block escape routes, making it difficult for people to evacuate the building^2^. If the furniture is lightweight, this problem can be overcome, as even if it topples, it can be easily removed from escape routes^1^. An average wardrobe weighs around 100 kg^3^ and can cause serious injuries if it falls on someone. The studies underscore the importance of safety measures and preventive strategies to overcome the risks associated with falls and falling objects across all age groups^4–8^.

In this study, furniture that is lighter than conventional furniture was produced. The term “earthquake-safe furniture” does not refer to furniture that can withstand heavy loads or resist earthquakes; instead, it refers to furniture that is lightweight and can be easily removed from escape routes in emergency situations. Additionally, the target of this furniture includes children’s furniture. Using lightweight furniture in kindergartens or children’s rooms will be safer^9–11^. The lightness of furniture offers various advantages in terms of ergonomics, safety and cost efficiency. When lightweight boards with reinforced cores are used in furniture production, superior structural integrity^12^ can be achieved while the overall weight of the furniture is reduced^13–16^. These lightweight materials not only reduce transportation costs by making furniture easier to move^13^ but also increase ergonomics by enabling easier assembly and disassembly for end users^13,17^. Additionally, the use of lightweight boards can enhance safety by offering sturdy yet easily portable furniture options, exemplified by lightweight tables featuring hardened foam bodies for structural support^18^. Additionally, the cost-effectiveness of lightweight furniture is highlighted by its potential to reduce production costs and increase market opportunities for manufacturers^19,20^. Overall, lightweight furniture presents an interesting case for improving ergonomics, safety and cost efficiency in the furniture industry.

**Fig. 1:**
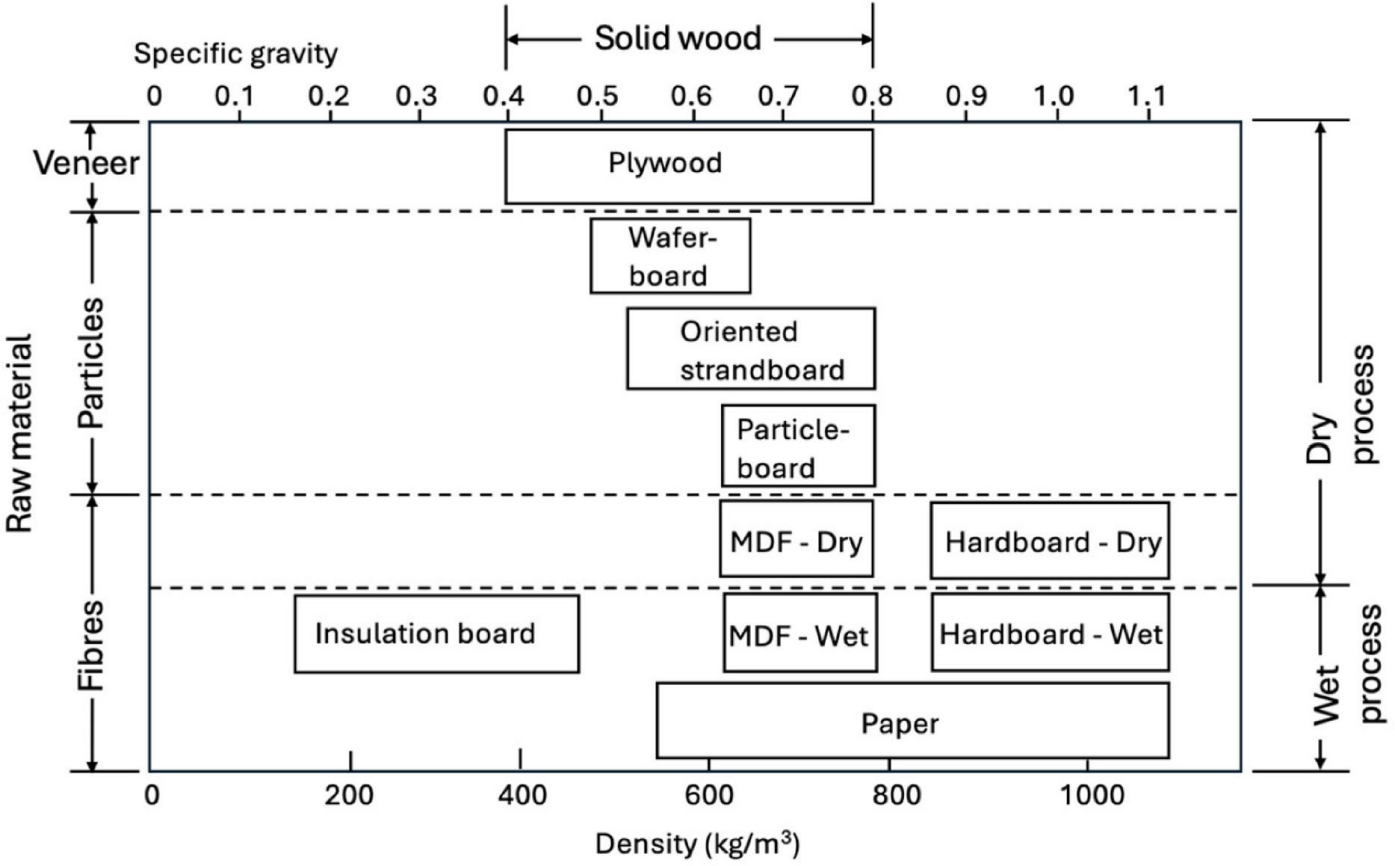
Classification of wood composites based on particle size, density, and production method^23,24^.

By promoting sustainable forestry and limiting deforestation, the use of lightweight panels also ensures long-term ecological benefits^21,22^. These panels thus play a crucial role in both environmental conservation and structural safety, making them a valuable material in sustainable construction practices. Wood industry wastes (wood particles and fibers) are used in the production of different wood composites. The most important of these are oriented strand board (OSB), MDF and three-layer PB. These boards have different density and chip geometry. Figure 1 shows the density ranges of the plates.

**Fig. 2:**
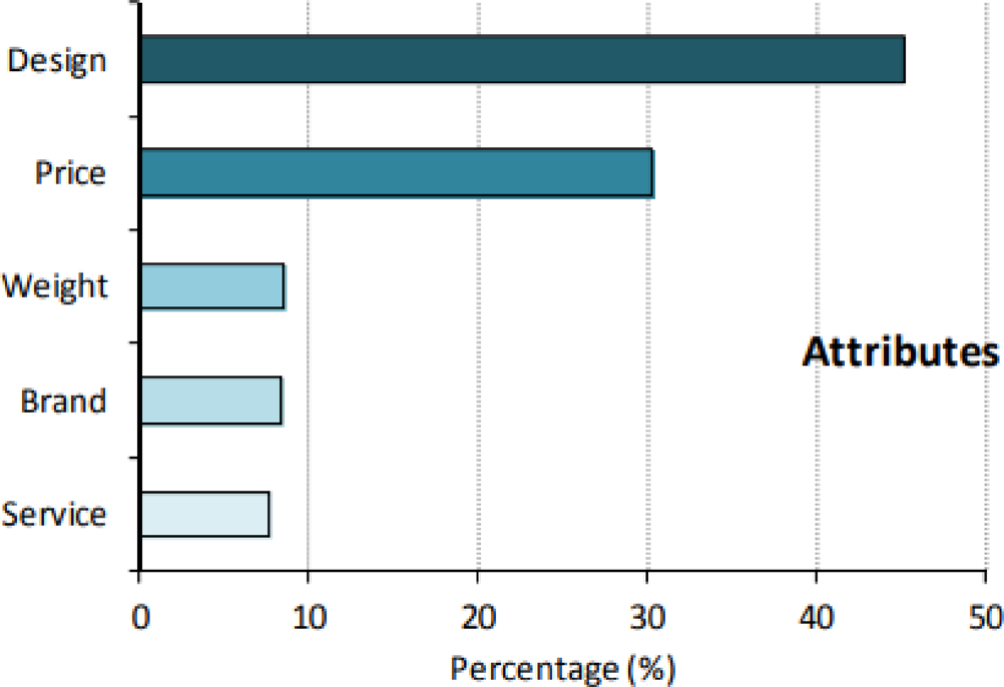
Furniture purchasing priorities^30^.

The weight reduction of wood-based panels is a critical consideration that varies depending on their final application. Over the years, strategies to reduce panel density have evolved, primarily categorized into three main approaches: technological advancements, material innovations, and the sandwich concept. Technological advancements optimize manufacturing processes to produce lighter panels without sacrificing performance^25,26^. Material selection focuses on using lightweight composites to achieve weight reduction while maintaining strength^27^. The sandwich concept incorporates lightweight cores between two face sheets, offering a balance between weight and mechanical properties^28,29^. Additionally, lightness is an important factor in customers’ furniture preferences (Fig. 2).

The wood sandwich panel examined in this study incorporates an XPS core, which is lightweight but combustible, potentially elevating fire risks. However, this limitation can be mitigated by employing alternative lightweight materials with enhanced fire resistance, including mineral-based cores or foams treated with fire-retardant agents. The primary goal of this study was to demonstrate that lightweight core materials (density 10–30 kg/m^3^) can be used in the middle layer to produce lightweight furniture suitable for everyday household use. While XPS was selected for this study, the concept can be extended to other materials, providing flexibility in balancing weight, cost, and fire performance for specific applications.

This study aims to design, produce, and standardize lightweight indoor furniture for many fields. The produced lightweight boards were compared with MDF and PB, which are the most used in furniture production. Thus, the usability of the produced lightweight (panel) boards in furniture production has been studied to be revealed comparatively. For this purpose, a bookshelf, wardrobe, dining table, and coffee table have been designed, produced, and standardized. These pieces of furniture do not contain any fabric parts. They are entirely made of wood and fastening components. In response to the increasing awareness and demand for earthquake safety, the findings and results obtained from this study will fill a significant gap in the indoor furniture industry and provide a solution that enhances public safety. This study not only ensures safety against injuries caused by furniture toppling during an earthquake but also contributes to the dissemination of innovative and sustainable practices in the furniture industry.

## Material and methods

### Materials

Light wood (300 kg/m^3^) sandwich panels (HM) were used to manufacture the light furniture. HMs were produced in the carpenter workshop by bonding MDFs to extruded polystyrene (XPS) with wood glue. Other materials, including screws, hinges, handles, drawer rails, furniture legs, were used to build the furniture components. In the production of HM, 10 mm thick XPS was used in the core layer (CL). The density of XPS was 10–15 kg/m^3^, specific heat was 1.3–1.7 kJ/kg.K, heat transfer coefficient was 0.028–0.036 W/m.K, compressive strength was 0.20–0.70 N/mm^2^, fire resistance was B1 and B2 (B1: Hardly flammable, B2: Normally flammable class) and vapor permeability resistance was 80–220. Medium density fiberboard (MDF) used in the surface layer (SL) was one side painted white color and the other side was unpainted. MDF was purchased from the local market in a size of 1700 mm × 2100 mm. Some properties MDF are given in Table 1. Water-based polyvinyl acetate glue (PVAc) was used to obtain the samples. PVAc polymers compose of vinyl acetate units, their appearance was white viscous liquid, density was 0.96 g/mL, solid content (%) was 41 ± 1, viscosity was 14,000 ± 1000 cps at 22 °C (Spindle No 6, 20 rpm), application quantity was 200 g/m^2^, water Resistance class was D2. All samples were prepared according to DIN EN 204^31^.

**Table 1:** Some technical properties of MDF which used in HM.

Properties	Standard	Unit	Scale
24-hour thickness swelling	TS 64EN 317	%	30
Modulus of rapture	TS 64EN 310	N/mm^2^	23
Modulus of elasticity	TS 64EN 310	N/mm^2^	2700
Internal bond	TS 64EN 319	N/mm^2^	0,7
Screw withdrawal resistance	TS 64EN 320	N	1000
Moisture content	EN 323	± 3%>	8
Density	EN 323	± 5%	850
Formaldehyde emission	EN ISO 12460-5^32^	mg/100 g.	≤ 8.0

### Preparation of samples

**Fig. 3:**
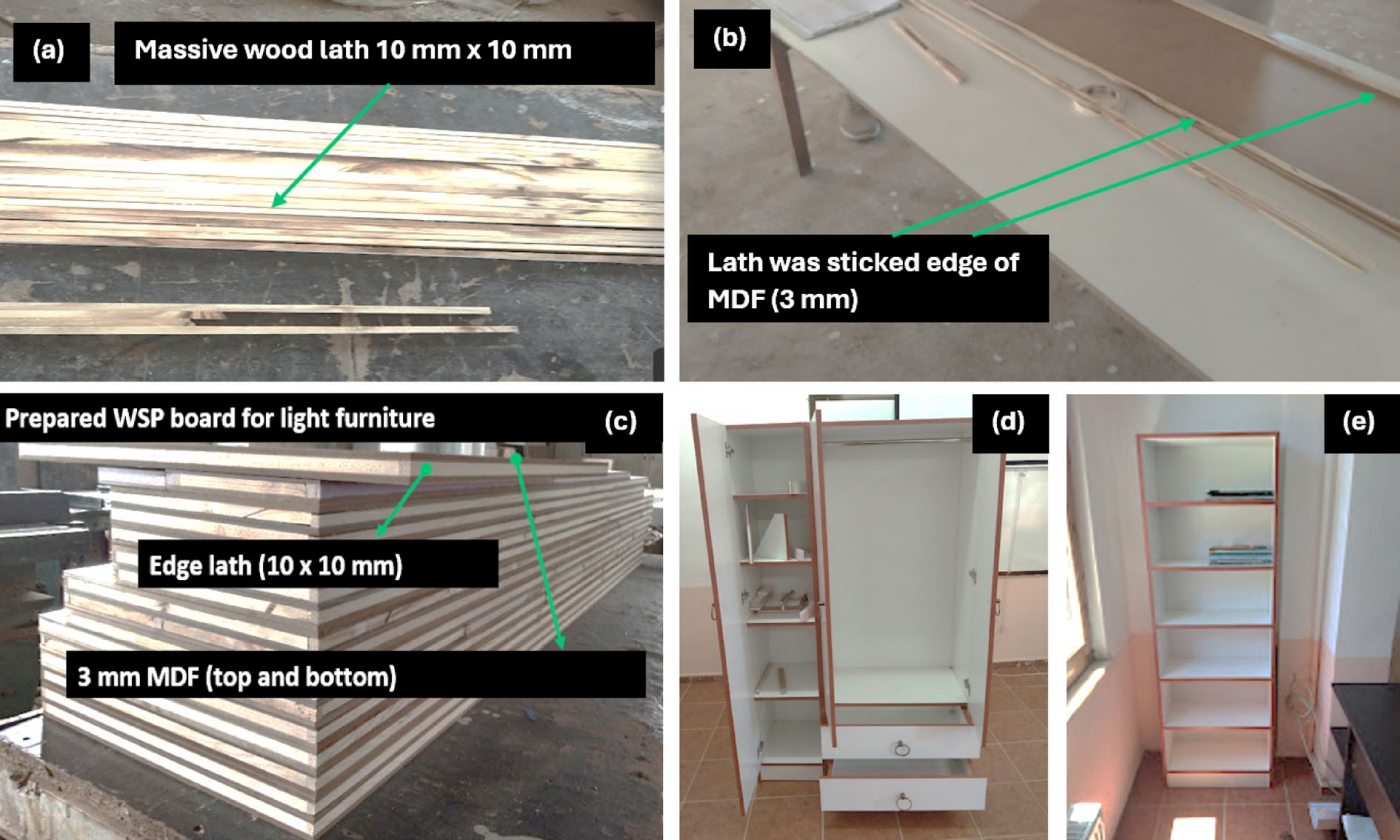
Manufacturing process of light furniture (**a**) cutting by diagonal saw, (**b**) edge lath montage, (**c**) prepared HM, (**d**) final wardrobe, (**e**) final bookcase.

The wood based furniture was designed on paper and its dimensions were assigned. Then, the dimensions of all wooden parts were determined, and the part numbers were written on them. A total of 4 furniture models, including (1) wardrobe, (2) bookcase, (3) dining table, (4) coffee table, were carried out. 10 mm × 10 mm Fir lath was glued to the edges of the boards to increase the screw holding resistance (Fig. 3a–c). Fir slats assembled in the form of a frame were placed on MDF (Fig. 3b). 10 mm XPS was placed in the middle of the lath in the size of the lath frame. XPS and lath surfaces were glued with a brush with 200 g/m^2^ and the same sized MDF was placed on them and pressed. For this, MDF with dimensions of 18 mm × 2100 mm × 2800 mm was laid on the ground for the pressing process. HM samples, whose gluing process was completed, were placed on the MDF on the ground and 45 boards (700 kg /m^3^) were placed on them to make them weigh and pressure was applied for 24 h. The HMs prepared according to their dimensions were taken to the assembly stage (Fig. 3d,e). 3.4 mm × 38 mm steel screws were used for assembly. Light weighted furniture was assembled, as seen in Fig. 3. To mechanical and physical analysis, 7 replicates for the lightweight furniture were used.

### Characterizations

**Fig. 4:**
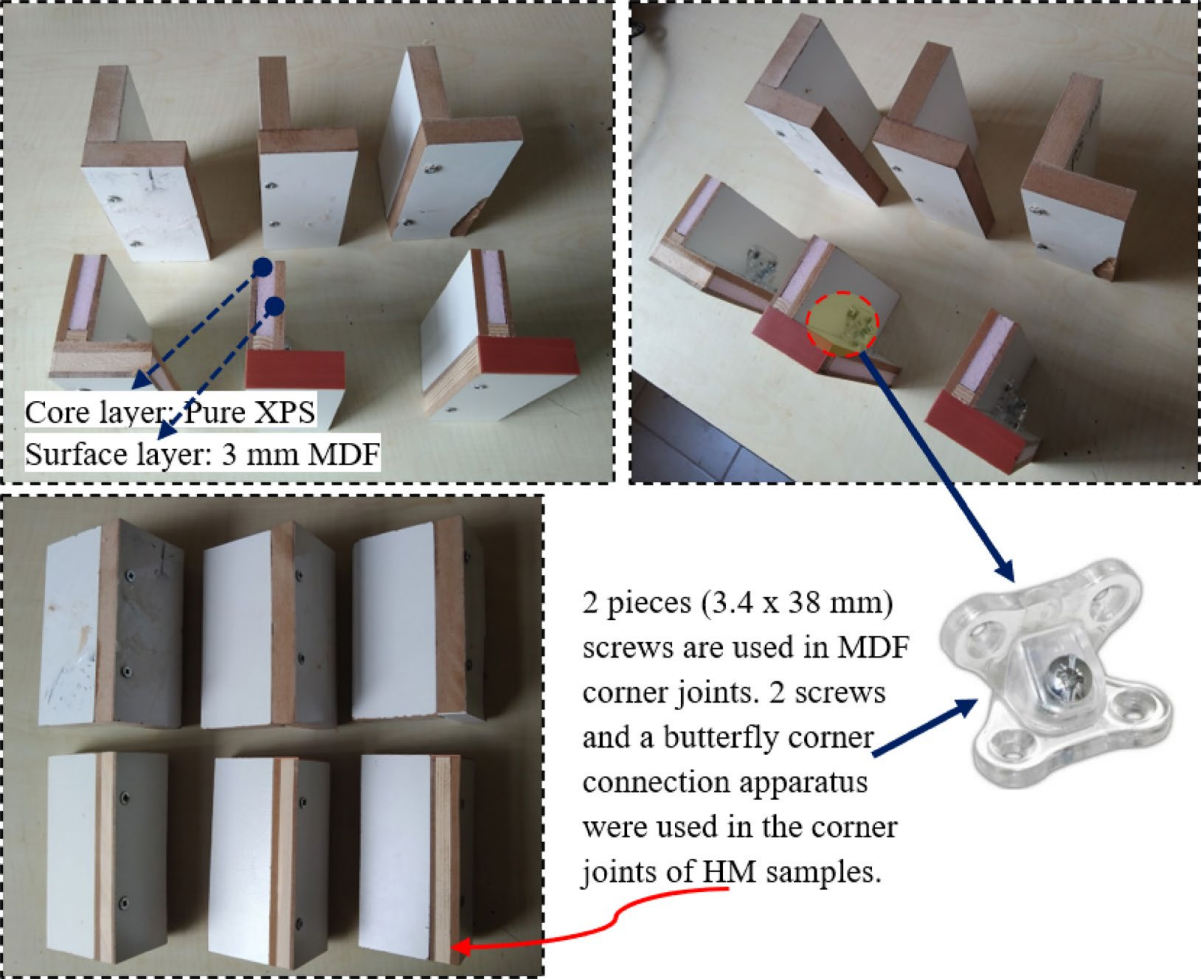
CJS strength analysis samples.

Test samples were prepared according to TS EN 325^33^ for mechanical and physical tests. Additionally, the previous test method in the literature was modified to determine to corner joint strength (CJS), and the CJS of the panels were evaluated according to the test methods^34–37^. This method was chosen for its simplicity and practicality. It allows testing using a standard mechanical testing device with a 5000 N capacity and provides results that are easy to interpret. In this method, two 100 mm × 70 mm HM pieces were joined with screws at a 90° angle (Fig. 4). The corner joint resistance was determined by applying a perpendicular load at a speed of 5 mm/min, and the maximum force recorded was used for the calculations.”

The modulus of rupture (MOR) test was performed on the specimens dimensioned with 410 mm × 50 mm × 18 mm according to TS EN 310^38^. The internal bond (IB) strength test was conducted based on TS EN 319^39^, using 50 mm × 50 mm specimens. Similarly, the screw withdrawal resistance (SR) test was carried out following TS EN 320^40^, also with 50 mm × 50 mm samples. All mechanical tests (CJS, MOR, IB, and SR) were conducted at a uniform loading speed of 5 mm/min, using a Zwick testing machine with a maximum capacity of 5000 N.

For physical properties, thickness swelling (TS) in accordance with TS EN 317 317^41^, water absorption (WA) in accordance with TS EN 322^42^, and density in accordance with TS EN 323^43^. The TS and WA tests were performed using a 10 L Memmert water bath, ensuring the specimens were fully submerged under the required conditions.

### Statistical analysis

Physical and mechanical analyses were performed for three groups of panels (MDF, HM, PB). The data were analyzed using the SPSS software package version PASW statistic18^44^. A one-way ANOVA was conducted, followed by Duncan’s multiple range test, to evaluate differences among the samples. The significance level was set at *P* ≤ 0.05 to determine statistical differences.

## Results and discussion

### Physical properties

Physical analysis results were given in Table 2. The MDF board varied between 750 and 800 kg/m^3^. MDF was the highest density. The HM density was around 450 kg/m^3^. This was the lowest density board and has the advantage of low costs and light weight. The density of PB varied between 600 and 650 kg/m^3^ (Table 2). It was lower than MDF, but higher than HM. HM was the most advantageous in this regard, as its low density provides an advantage in terms of low costs. In terms of the TS, MDF swelled approximately 2% after 2 h and approximately 6% after 24 h. HM nearly 0% swelled after 2 h and approximately 1% swelled after 24 h and was the board with the lowest swelling rate. PB approximately 6% swelled after 2 h and approximately 14% swelled after 24 h and was the board with the highest swelling rate (Table 2). Since low swelling is a preferred feature, HM board was the most advantageous in this regard.

**Table 2:** Physical analysis results of samples.

Samples	TS-2 h (%)	TS-24 h (%)	WA-2 h (%)	WA-24 h (%)	Density (kg/m^3^)
MDF	0.4 (± 0.35) ^a^ A ^b^	4.2 (± 0.48) A	6.3 (± 2.2) A	15 (± 3.2) A	785 (± 18) C
HM	0.4 (± 0.08) A	3.5 (± 0.82) A	5.2 (± 1.1) A	18 (± 3.5) A	443 (± 6) A
PB	8 (± 0.72) B	13 (± 2.37) B	97 (± 4.3) B	101 (± 5.8) B	515 (± 39) B

^a^ Standard deviation.

^b^ Mean values with the same letter within the same column are not significantly different (*p* > 0.05).

**Fig. 5:**
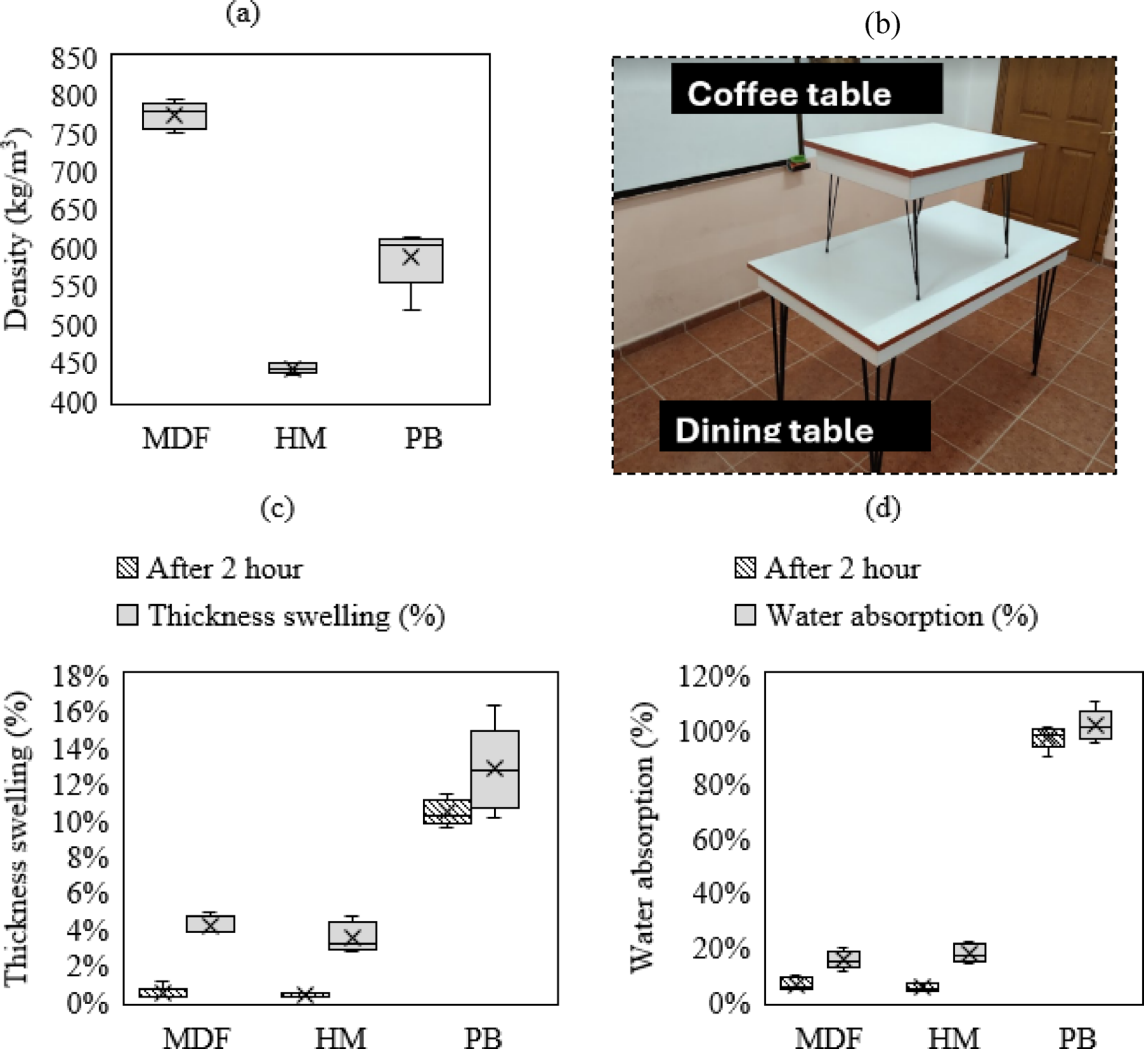
Physical properties of board samples, (**a**) density, (**b**) prepared furniture, (**c**) thickness swelling, (**d**) water absorption.

In terms of water absorption, MDF absorbed approximately 10% water after 2 h and approximately 25% after 24 h. HM absorbed approximately 20% water after 2 h and approximately 25% water after 24 h. PB absorbed approximately 50% water after 2 h and approximately 100% after 24 h and had the highest WA rate (Table 2). Since low WA is a preferred feature, MDF and HM boards were more advantageous than PB boards. Although the WA between MDF and HM have similar, HM is slightly more advantageous in the WA. In general, HM board was the most advantageous board in terms of density, TS and WA. It provides advantages in terms of cost and lightness with its low density. In addition, it was the board with the lowest TS and WA, which made HM the most suitable material in terms of physical properties. MDF, on the other hand, may be higher in cost due to its higher density, but its TS and WA were moderate. MDF had less advantage over HM but still outperformed PB. PB, was the board with the worst performance in terms of density, TS and WA. For this reason, this board type may be less preferred by manufacturers than others. According to the analyzed data, HM board was the best performing board type.

Considering the advantages of lightweight furniture (Fig. 5b) over heavier alternatives, such furniture is particularly preferred for its portability, cost-effectiveness, and sustainability. However, improving the physical properties (Fig. 5a,c,d) of lightweight materials is crucial for expanding their potential applications. Also, enhancing mechanical strength, impact resistance, and load-bearing capacity can make light weight furniture more widely accepted in residential and commercial settings^45^. This is especially relevant for office furniture, portable systems, and temporary spaces^19^.

This study produced lightweight panels composed of an XPS core layer and MDF surface layers, which were used to manufacture household furniture (cabinets, bookshelves, tables, and coffee tables). This aligns with previous research by focusing on improving strength-to-weight ratios and promoting innovative material solutions for furniture production.

Differently, this study emphasizes the integration of lightweight XPS panels with traditional MDF surfaces, offering a unique combination of structural integrity, functionality, and production efficiency. This approach provides a cost-effective and practical alternative to advanced composites, while also addressing sustainability and design flexibility in the context of household furniture manufacturing.

### Mechanical properties

**Fig. 6:**
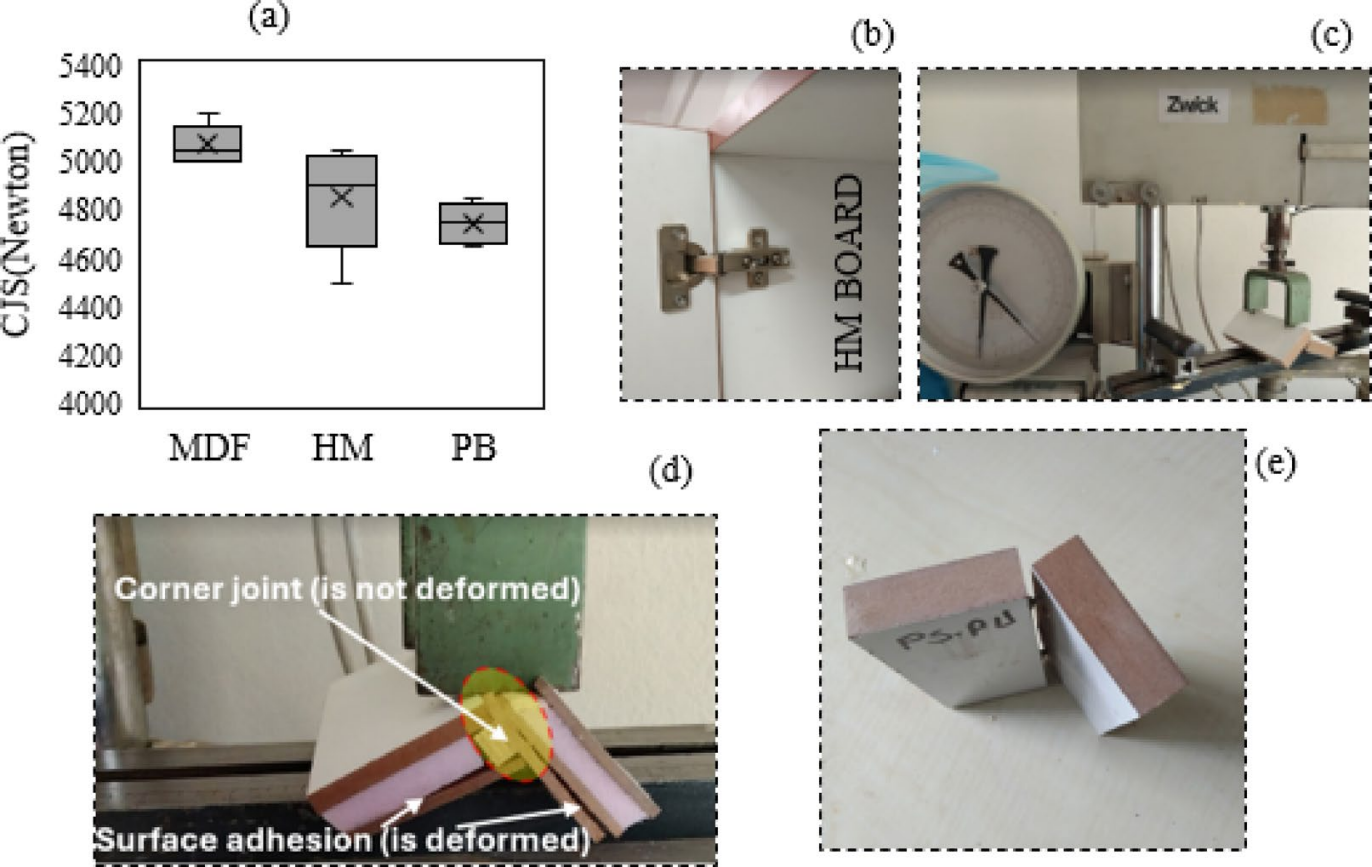
CJS strength of board samples (**a**), light furniture CJS (**b**), CJS test (**c**), HM CJS analysis sample (**d**) MDF CJS analysis sample (**e**).

Mechanical analysis results of samples were given in Table 3. As seen in Table 3, MDF material had the highest corner joint strength (Fig. 6c). The average strength value was around 5100 Newtons and was in a narrow distribution range. This demonstrated the high and consistent performance of MDF (Fig. 6e). These features of MDF support its preference in construction and furniture applications^46^.

**Table 3:** Mechanical analysis results of samples.

Sample	MOR (*N*/mm^2^)	IB (*N*/mm^2^)	SR (Newton)	CJS (Newton)
MDF	22.3 (± 2.5)^a^ C	0.7 (± 0.03) B	3640 (± 207) C	5070 (± 83) B
HM	8.7 (± 0.7) A^b^	1.1 (± 0.08) C	2540 (± 230) A	4850 (± 217) A
PB	12.7 (± 0.8) B	0.35 (± 0.02) A	3062 (± 63) B	4746 (± 82) A

^a^ Standard deviation.

^b^ Mean values with the same letter within the same column are not significantly different (*p* > 0.05).

HM sample had a wider strength distribution. While the median was approximately 4900 Newtons, the strength was between 4600 and 5150 Newtons (Fig. 6a). This variability indicated fluctuations in HM’s performance. This property of HM requires careful consideration in specific applications. The CJS of the HM board was quite strong. However, the same could not be said for the adhesive surface between MDF and XPS (Fig. 6d). It is obvious that the mechanical properties would be better if a stronger adhesive was used instead of PVAc glue in the production of HM boards. The evaluation of sample failures indicated that there were no issues related to the adhesion between the wood and the glue. Instead, the limitations in mechanical performance were primarily associated with the intrinsic bonding strength of the adhesive itself. Employing an adhesive with higher internal cohesion would therefore contribute to improved overall durability of the HM boards. This would not only facilitate the assembly of furniture components, such as hinges (Fig. 6b), but also significantly enhance connection strength. Visual evidence supporting this assessment is provided through images of sample failures. PB sample exhibited the lowest CJS. The average strength value was 4700 Newtons, and the distribution range was between 4650 and 4750 Newtons. PB’s low strength and inconsistent performance indicate its suitability for use in less critical applications^47^.

While MDF stood out with its high strength and consistency, HM material could show high performance under certain conditions but had a variable structure. PB material can be used in limited application areas due to its low strength. These findings provide important guidance in material selection and contribute to making the right material choice in the furniture industry.

**Fig. 7:**
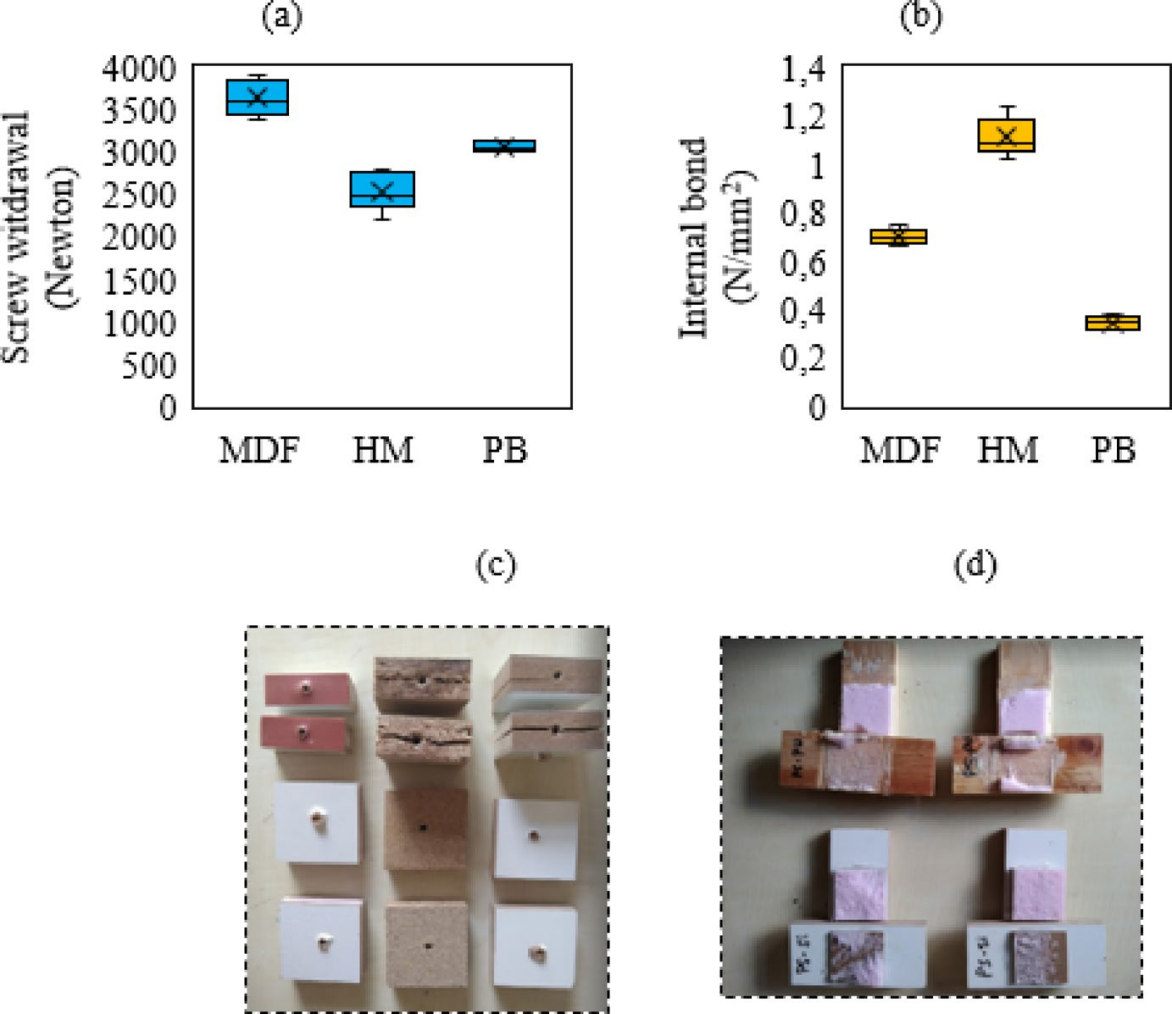
(**a**) Screw withdrawal resistance of samples, (**b**) internal bond, (**c**) screw withdrawal analysis samples, (**d**) HM internal bond analysis samples.

SR and IB strengths of MDF, HM and PB samples were given in Table 3. The SR strength of MDF was the highest, approximately 3500 N. The SR of HM and PB were close to each other, HM was approximately 2500 N and PB was approximately 3000 N. These results showed that the SR capacity of MDF was superior to the other two samples. The high density and homogeneous structure of MDF were the main reasons for the high SR strength. These results are consistent with other studies in the literature^48,49^. The high IB strength of MDF is achieved thanks to the good bonding of the fibers and the homogeneous distribution of the resin. The lower IB strengths PB are due to the differences in production processes used in these materials. These results are also consistent with the findings in the literature^50,51^. It can be said that HM was superior to MDF and PB in terms of IB strength. In the assembly of lightweight furniture made with HM, supporting fasteners (butterfly screws) can be used together with screws. Thus, corner joints are strengthened and can be more durable like MDF. Graphical diagrams and photographs of the screw withdrawal and internal bond examples are given in Fig. 7a–d.

**Fig. 8:**
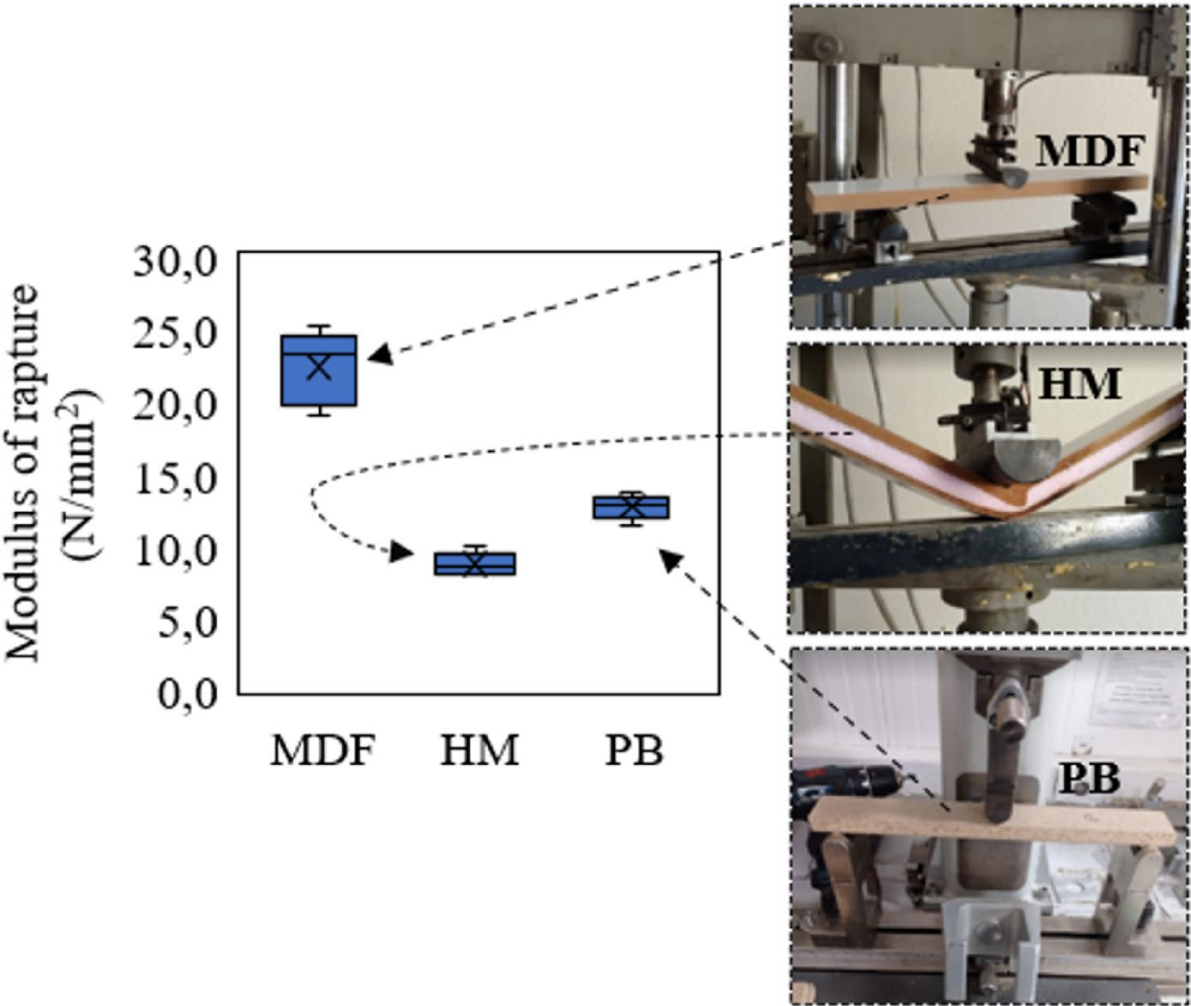
Modulus of rapture of samples.

The MOR strength of MDF was quite high compared to the other two samples. This showed that MDF is more durable (Fig. 8) and can carry more loads. This high durability is due to the good bonding of fibers in MDF and the homogeneous distribution of resin^50,51^. The MOR of HM is lower than MDF and PB. The MOR strength of HM was the lowest. This showed that HM is less durable and can carry less loads than MDF and PB. This problem can be eliminated if thinner and denser (e.g. compact laminate) is used as coating material on XPS. In this way, both the density does not increase and the MOR is increased. A graphical diagram of the MOR strength is given in Fig. 8.

## Conclusions

In this study, in light of all this data, lightweight furniture (approximately half the weight of traditional furniture) was produced. The cost of the wood sandwich panel, consisting of a polystyrene core layer and 3 mm MDF surface layers, was calculated for January 2025. The price of 15-density XPS is approximately $1–1.5 per square meter, and the cost of 3 mm MDF for both the top and bottom surfaces is around $2–2.5. Therefore, the total cost of a 16 mm wood sandwich panel is estimated to be $3–4 per square meter. In comparison, the cost of a 16 mm MDF panel is approximately $6.5–7 per square meter. This demonstrates that the cost of the wood sandwich panel (HM) is 50–70% lower than that of a standard MDF panel.

The main reason of why the furniture is lightweight is that the furniture was manufactured light board. For this, a special board (HM) was produced in the carpenter workshop. The surface of the HM was MDF and its core was XPS. The furniture strength produced with HM was compared with those produced with PB and MDF. According to the results obtained:

- HM board was considerably superior to MDF and PB boards in terms of water absorption (21%) and the in thickness swelling (20% better).
- HM board was 77% lighter than MDF board and 16% lighter than PB board.
- HM’s CJS strength (4850 N) was close to PB (4746 N). (If stronger adhesive was used in HM production, CJS strength could be made superior to MDF (5070 N) and PB. Thus, HM furniture becomes more durable than traditional furniture.
- HM’s IB strength (1.1 N/mm^2^) was higher than MDF (0.7 N/mm^2^) and PB (0.35 N) depending on the adhesive strength of the glue.
- HM’s SR strength (2540 N) was lower than MDF (3640 N) and PB (3062 N) due to the density of XPS in the core layer. This was a problem during the assembly of the furniture. This problem was solved by adding a 10 mm × 10 mm wooden lath to the area to be screwed.
- HM’s MOR strength (8.7 N/mm^2^) was lower than PB (12.7 N/mm^2^) and MDF (22.3 N/mm^2^) due to the MOR strength of XPS in the core layer. This was the most difficult problem to overcome. This problem can be overcome by using thinner and denser material (like compact laminate) on the surface layer.

PB and MDF panels are predominantly used for the production of home and office furniture. In this study, the potential of utilizing lightweight and cost-effective wood sandwich panels (HM) for manufacturing such furniture was investigated. The findings demonstrated that furniture produced with HM panels can achieve durability levels comparable to traditional home and office furniture.

It is suggested that further research on this topic could lead to the development of furniture that is more cost-effective, consumes less wood, and is significantly lighter. Such weight reduction would provide additional benefits, including minimizing the risk of injuries during earthquakes in case of furniture toppling and facilitating the removal of furniture obstructing escape routes. These features could contribute to safer and more sustainable furniture solutions for homes and offices.

## Data Availability

The datasets used and/or analysed during the current study available from the corresponding author on reasonable request.

## Abbreviations

HM: Light wood sandwich panel
MDF: Medium density fiberboard
PB: Particleboard
XPS: Extruded polystyrene
CL: Core layer
SL: Surface layer
PVAc: Water-based polyvinyl acetate glue
CJS: Corner joints strength
MOR: Modulus of rapture
MOE: Modulus of elasticity
IB: Internal bond
SR: Screw withdrawal resistance
TS: Thickness of swelling
WA: Water absorption
